# A working memory dependent dual process model of the testing effect

**DOI:** 10.1038/s41539-024-00268-0

**Published:** 2024-09-09

**Authors:** Yicong Zheng, Aike Shi, Xiaonan L. Liu

**Affiliations:** 1 Department of Psychology, University of California, Davis, CA USA; 2 Department of Psychology, The Chinese University of Hong Kong, Shatin, Hong Kong

**Keywords:** Human behaviour, Human behaviour, Cognitive control

## Abstract

This Perspective article expands on a working memory-dependent dual-process model, originally proposed by Zheng et al.^1^, to elucidate individual differences in the testing effect. This model posits that the testing effect comprises two processes: retrieval-attempt and post-retrieval re-encoding. We substantiate this model with empirical evidence and propose future research. This model invites further studies on the trade-off between testing benefits and WM demands, facilitating the development of personalized educational practices.

## Introduction

Imagine a scenario where a group of students are taking the same quiz as a practice for their final exam. After the quiz, some students might get a deeper understanding of the material, while some might find the exam more challenging and didn’t benefit from it as much as others. It is always intriguing to educators why individuals gain massively different outcomes given the same instructions, assignments, and exams. Here, we focus on individual differences in one well-established phenomenon in education—the Testing Effect. The existing evidence of the testing effect traces back to as early as 1917 when Gates found that recitation produces a better memory outcome than “read-only”^2^. That is, when people are prompted to reproduce learned materials, testing benefits memory more than re-learning materials^3–5^. Numerous empirical studies have confirmed the promotion of learning by various forms of testing in both laboratories^6–8^ and classroom environments^9–12^. Moreover, researchers have conducted a large number of studies to explore the influencing factors of the testing effect, such as the form of testing^13–15^, attention allocation^16^, and whether there is feedback after the testing^17^.

Understanding individual differences in the testing effect is particularly important in education (quoting Confucius, “teach students in accordance with their aptitude”). However, an agreement on how individual differences affect the benefits of testing has not been reached. For instance, previous research has found that tests are instrumental in improving the performance of later recall irrespective of prior knowledge^18–20^ and personality characteristics^21^, whereas people with relatively low-level intelligence can fail to benefit from taking retrieval practice when the task is difficult^22,23^ and students with higher academic achievements benefit more from retrieval practice^24^. Moreover, the controversy over the applicability of the testing effect across different individuals reached its peak when it comes to working memory capacity (WMC) (Table 1). Some studies showed that people with lower WMC benefit more from testing^25,26^, some studies showed the opposite pattern that people with higher WMC benefit more from testing^1^, and some studies did not find a relationship between WMC and the testing effect^27–29^.

**Table 1:** Studies that examined the effects of individual differences in working memory capacity on the testing effect

Study	Individual difference factors	Stimuli	Feedback	Main findings
Agarwal et al. ^25^	WMC	General knowledge facts	Manipulated	Participants with lower WMC showed larger testing effect with feedback
Bertilsson et al. ^21^	WMC Personality	Swahili word pairs	Yes	WMC did not predict the testing effect
Bertilsson et al. ^27^	WMC Personality	Swahili word pairs	Yes	WMC did not predict the testing effect
Brewer & Unsworth^28^	WMC Gf Intelligence	English word pairs	Yes	WMC did not predict the testing effect
Minear et al. ^22^	WMC Gf Intelligence	Swahili -English word pairs	Yes	WMC did not predict the testing effect
Tse & Pu^26^	WMC Test anxiety	Swahili -English word pairs	No	Interaction between trait test anxiety and WMC: the testing effect decreased with test anxiety for participants with lower WMC, but it did not correlate with test anxiety for those with higher WMC
Tse et al. ^43^	WMC Test Anxiety	General knowledge facts	No	No interaction between WMC and trait test anxiety in the testing effect.
Wiklund-Hörnqvist et al. ^29^	WMC	Concepts in a Psychology course	Yes	WMC did not predict the testing effect
Zheng et al. ^1^	WMC	Image - word pairs	No	Consistent testing effect in participants with higher WMC. Testing effect only emerged in participants with lower WMC when learning low WM demanding stimuli

## Current theories of the testing effect do not consider individual differences

While multiple theoretical explanations of the testing effect have been proposed, these theories do not fully explain why some people benefit more from testing than others. Prominent theories of the testing effect can be broadly divided into two categories. The first category of theories focuses on the memory retrieval process itself, that is, the process of trying to recall information from memory. One such theory is the Transfer-Appropriate Process account^30^. Supposing successful recall depends on the similarity between the encoding and retrieval processes, the TAP account proposed that the insertion of tests between the initial study and the final test makes it possible for people to access the contents more readily in a later test because of the high similarity between the practice test and the final test. Another notable theory is the Elaborative Retrieval Theory^31^, which states that when people are tested, they must actively search their memory for the answer. This process of extracting information from memory strengthens existing retrieval pathways and may also create new ones (i.e., mediators^32^). As a result, testing is a more effective learning method than simply rereading material. Additionally, the Retrieval Effort Hypothesis^4^, grounded in the Desirable Difficulty Hypothesis^33^, argues that retrieval is beneficial because it imposes appropriate difficulty and requires substantial cognitive resources. This additional investment enhances memory retention. Moreover, the Episodic Context Account emphasizes that testing enables subjects to recall both the original learning context and the context of the retrieval practice phase. This dual-context memory allows for a broader search range during final tests, thereby improving recall success, as noted by Lehman et al. ^34^.

The other category of theories focuses on information processing after successful memory retrieval, that is, the additional processing of the obtained answer. One example is the Reconsolidation Account^35,36^, which argues that what promotes memory retention is not the retrieval process itself, but the reconsolidation process after information is successfully retrieved^37^. During this state, information can be either strengthened or interfered with^38,39^. When participants correctly retrieve the target or when correct feedback is provided after retrieval, memories are more likely to be strengthened due to the reexposure to the correct targets. In other words, the role of retrieval is to put memory into an unstable state, and the process after retrieval strengthens memories.

In summary, although these theories can explain many phenomena associated with testing, they cannot explain how people with different WMCs benefit differently from testing and predict when testing is the optimal learning approach.

## A working memory dependent dual-process model

**Fig. 1:**
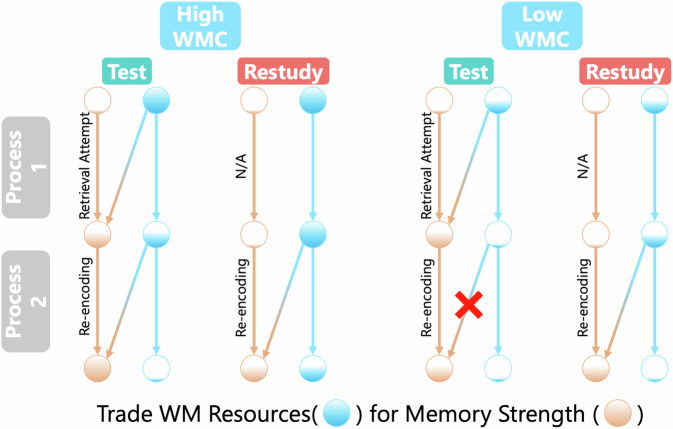
The dual-process model of individual differences in the testing effect: a comparison between high and low Working Memory Capacity (WMC) groups. The figure illustrates a cued recall task, contrasting successful retrieval practice (Test) with Restudy trials for individuals with varying WMC. Both retrieval and re-encoding processes consume available WM resources (depicted in blue), leading to an increase in memory strength (depicted in yellow). In individuals with high WMC, the Test condition offers greater benefits over Restudy due to sufficient working memory resources. Conversely, for those with low WMC, Restudy may be as effective as testing—or even outperforms testing, when WM resources are not sufficient for the re-encoding process.

In this section, we elaborate on a working memory-dependent dual-process model of the testing effect designed to elucidate individual differences in WMC. This model was first introduced by Zheng et al.^1^ and focuses on how two temporally ordered processes, *retrieval attempt*, and *post-retrieval re-encoding*, interact with available WM resources that result in individual differences in the testing effect (Fig. 1). The “retrieval attempt” process involves the initiation of a search to find the answer to a test question. It not only serves the current retrieval but also further strengthens memories, consistent with theories such as the Elaborative Retrieval Theory. The “post-retrieval re-encoding” process pertains to the reiteration of the correct answer in WM after a successful retrieval, aligning with theories like the Reconsolidation Account.

The effectiveness of these two processes in memory strengthening is modulated by the available WM resources during the task. These resources are intrinsically linked to individual WMC and the WM demands of the material being memorized. In a metaphorical sense, both processes draw from the same pool of WM resources, enabling a trade-off between WM expenditure and memory fortification.

The idea of trading WM resources for memory strengthening has been quantitatively implemented in a modified Source of Activation Confusion (SAC) model^1,40,41^. SAC is a classic localist memory model positing spread activation within a network composed of “concept” and “episode” nodes connected by associative edges. Memory strength is delineated by base-level strength and activation level, representing the quality of the stored memory and its activation status during retrieval, respectively. Nodes activated by proactive retrieval or spreading activation from connected nodes will exhibit a high temporary activation level, thereby increasing the base-level strength.

Additionally, the SAC model quantifies the interaction between long-term memory and WM, by proposing a limited pool of WM resources. Individuals with higher WMC have greater WM resources available for diverse tasks, while those with lower WMC have fewer resources at their disposal. Node operations, including encoding and retrieval, consume these WM resources, which are subsequently replenished over time.

It is important to clarify that our model does not posit that individuals with low WMC are categorically unable to benefit from testing. Instead, the model integrates another crucial determinant: the working memory (WM) demands associated with the material being learned. According to the SAC framework, the familiarity of a memory node impacts the amount of WM resources consumed during cognitive operations such as encoding and retrieval. For instance, the process of encoding or retrieving a novel item would deplete more WM resources than would familiar items. Therefore, the model predicts that individuals with low WMC could experience limited or even negligible benefits from testing when learning materials that are unfamiliar and demanding in terms of WM resources. This is because the act of attempting to retrieve unfamiliar information could disproportionately tax their limited WM capacity, leaving insufficient resources for re-encoding. Importantly, the model predicts that they could still benefit from the testing effect when materials require WM resources that could be covered by their WMC.

## Behavioral evidence supporting the model

Recent research by Zheng et al.^1^ provides compelling behavioral evidence that supports this dual-process model, examining the interplay between WMC and WM demands in the testing effect. Notably, the study found that individuals with high WMC exhibited the testing effect irrespective of stimulus frequency. Conversely, for individuals with low WMC, testing enhanced retention only for high-frequency stimuli; low-frequency stimuli actually yielded a negative testing effect, where restudy proved more effective than testing.

These findings corroborate the core hypothesis of our model concerning the dynamic between available WM resources and the WM demands of the task. For individuals with higher WMC, sufficient WM resources facilitated both the retrieval attempt and post-retrieval re-encoding processes, even for unfamiliar or low-frequency stimuli. However, for those with lower WMC, attempting to retrieve unfamiliar or low-frequency stimuli exhausted WM resources, preventing effective re-encoding.

Noteworthy, one potential concern with the results of Zheng et al.^1^ is that the observed interaction between WMC and WM demands may, in fact, be due to insufficient encoding of unfamiliar material by individuals with lower WMC, which leads to lower retrieval practice accuracy, rather than insufficient working memory resources during retrieval practice. We find this alternative hypothesis implausible for three reasons. First, in Zheng et al.^1^, this potential confounding was controlled by applying the same learning criteria for low- and high-frequency stimuli and regressing out the accuracy ratio between low- and high-frequency stimuli. Second, additional analyses of the data from Zheng et al. ^1^ by selecting only trials that were correctly retrieved during retrieval practice showed a similar pattern as including all trials. Third, and more critically, one recent study directly addressed this alternative hypothesis through a series of thorough experiments^42^. This study found no causal link between retrieval success and the magnitude of the testing effect. In other words, regardless of retrieval practice performance, the magnitudes of the testing effect were consistent. This study indicates that observed correlations between retrieval practice performance and the testing effect magnitudes are likely due to individual differences rather than experimental conditions.

Additional support for this model is derived from other behavioral studies. A cross-study comparison from the same research group indicates that the language in which learning materials are presented can influence the impact of individual differences on the testing effect. Tse & Pu^26^ identified an interaction between trait test anxiety and WMC, where individuals with lower WMC experienced a reduced testing effect when trait test anxiety was high. This effect was not observed in individuals with higher WMC, suggesting that those with higher WMC are less vulnerable to the negative influences of trait test anxiety. However, a follow-up study by Tse et al. ^43^ did not find a significant interaction between these variables using a similar design. A notable difference between the two studies was the language of the learning materials; the earlier study used materials in a foreign language, which likely imposed higher working memory demands than the materials in the participant’s native language used in the later study by Tse et al. ^43^.

Furthermore, the provision of feedback during retrieval practice appears to modulate the role of individual differences in the testing effect. Notably, Agarwal et al. ^25^ observed that participants with lower WMC experienced a more pronounced testing effect compared to those with higher WMC. However, this pattern was specific to the condition where feedback was provided after each retrieval practice trial. Additionally, a series of studies examining the relationship between WMC and the testing effect found no disadvantage for individuals with low WMC in terms of the magnitudes of the testing effect when feedback was provided immediately after each trial (e.g., Bertilsson et al. ^21^; Minear et al. ^22^; Table 1). These findings indicate that providing feedback and presenting the correct answer after retrieval can reduce the working memory load for participants with low WMC. By eliminating the need to maintain the retrieved answer in working memory during the post-retrieval re-encoding process, resources are freed up for memory strengthening, allowing individuals with lower WMC to benefit from testing in a manner similar to their higher WMC counterparts.

Relatedly, Tse et al. ^44^ discovered that, in the absence of feedback, older individuals demonstrated a negative testing effect, where restudy surpassed retrieval practice in effectiveness. However, a positive testing effect emerged when feedback was provided. This pattern is consistent across several studies, indicating that the testing effect diminishes in older adults without feedback post-retrieval, yet with feedback, older adults exhibit a comparable testing effect to that of younger adults^45–48^. Considering the well-documented decline in WMC among the elderly^49,50^, it is reasonable to hypothesize that WMC contributes to the observed interplay between age, feedback, and the testing effect. However, these studies involving aged groups should be interpreted cautiously. They provide only complementary insights into our model rather than direct evidence. The complex interplay of multiple declining functions with age means that causal relationships cannot be straightforwardly derived from these observations.

## Neuroimaging evidence supporting the model

In this section, we present evidence coming from neuroimaging studies that support the two main components of the proposed model: (1) The testing effect comprises two processes, retrieval attempt and post-retrieval re-encoding; (2) These two processes consume WM resources from the same pool.

A substantial body of neuroimaging research supports the dual-process aspect of our model. For example, fMRI studies that have applied subsequent memory analyses to retrieval trials^51,52^ identified that regions in the left hemisphere associated with successful encoding also predicted future retention following testing. However, unique right hemisphere regions specifically predicted retention only in testing trials. These findings suggest that the act of testing engages both a process akin to encoding and a process that is unique to retrieval practice. The spatially distinct neural correlates corroborate our proposed separation between the retrieval attempt and post-retrieval re-encoding processes. Retrieval attempts may predominantly engage regions in the right hemisphere, whereas re-encoding likely activates more left-lateralized areas that are also involved in encoding. Similarly, Wang & Yang^53^ reported distinct involvement of the salience and executive control networks (S-ECN) and the default mode network in error monitoring during retrieval attempts and the maintenance of correct answers during post-retrieval re-encoding, respectively.

As the two processes unfold sequentially, EEG/ERP studies, with their high temporal resolution, may offer more direct evidence supporting this model. Indeed, specific time windows of ERP components during retrieval practice have been associated with the two processes. For example, Liu et al. ^54^ revealed that an ERP component occurring between 400 and 700 ms after the presentation of retrieval cues predicted both current and subsequent retrieval success, potentially reflecting the elaboration and strengthening that occur during retrieval attempts. Furthermore, a later component between 700 and 1000 ms was associated solely with subsequent retention, serving as a neural marker for re-encoding after a successful retrieval. Additional studies have identified temporally distinct ERP components that align with those found by Liu et al. ^54^. For instance, Bridge and Paller^55^ found that, in an object-location association task, ERP amplitudes during the 400–700 ms window were correlated with the accuracy of the recalled location, while the 700–1000 ms window was correlated with performance on a subsequent test. Similarly, Bai et al. ^56^ found that the late component (700–1000 ms) predicted future, but not current, test accuracy. Moreover, Bencze et al. ^57^ observed a subsequent memory effect in two ERP components during retrieval practice: one from 500 to 700 ms and another from 700 to 1000 ms. These findings were interpreted to suggest that the earlier component is associated with episodic recollection, and the later component relates to post-retrieval evaluative processes.

Besides support for the two-process component in the testing effect, neural evidence also supports the assumption that both retrieval attempt and post-retrieval re-encoding consume the same pool of WM resources, a concept rooted in the original SAC model^40^. Lee et al. ^58^ demonstrated that both episodic memory encoding and retrieval activate similar regions of the lateral prefrontal cortex, challenging the functional-asymmetry model that suggests these processes use distinct WM resource pools. Further supporting this notion, Hsieh and Ranganath^59^ found that frontal midline theta activity is involved in both WM and episodic memory encoding and retrieval. This finding underscores the overlapping nature of the cognitive processes underlying these processes. Additionally, Rugg et al. ^60^ showed that successful recollection necessitates the engagement of processes involved in both encoding and retrieval, implicating the same cortical regions. This overlap highlights the interconnectedness of memory processes. Melrose et al. ^61^ further emphasized this connection by demonstrating that prefrontal cortex activity during WM tasks supports long-term memory learning and recall. Collectively, these studies indicate a significant overlap between WM and long-term memory encoding and retrieval, reinforcing the idea that both retrieval and re-encoding in the testing effect draw from a common pool of WM resources.

While the aforementioned studies provide substantial neural evidence supporting the assumption that both retrieval and post-retrieval re-encoding processes draw from a common pool of WM resources, it is important to note that this evidence remains indirect. Direct empirical validation of this assumption is still lacking. Future research should focus on designing paradigms that directly test the competition between encoding and retrieval tasks for working memory resources.

## Relationship with existing theories

It’s worth noting that although our model proposes a dual-process framework, it does not inherently conflict with existing theories of the testing effect that focus on one of the two processes. Mechanisms like semantic elaboration^31^, mediator effectiveness^32^, transfer-appropriate process^30^, and reconsolidation^35,36^ may still play a role in further explaining under which circumstances each process is beneficial.

Our model coincides with the Reconsolidation Account^35,36^, in which both models acknowledge the existence of two processes during the testing effect: the retrieval process per se and the post-retrieval process. However, our model diverges from the reconsolidation theory as described by Finn^35^ and Finn & Roediger^36^. The reconsolidation account focuses on the post-retrieval process, arguing that the retrieval practice marks memories as entering a “labile state”, making them susceptible to change. In other words, what actually strengthens memory is the post-retrieval process. However, our model states that both the retrieval attempt and post-retrieval re-encoding processes may strengthen memory. While the first process is guaranteed to strengthen memory, the second process’s strength depends on whether the first process consumes all the available working memory resources.

Moreover, our model aligns with the desirable difficulty framework^33^ in learning, which posits that effective learning necessitates a significant yet manageable level of effort. According to our model, when a task significantly challenges an individual’s WMC, the “difficulty” generated by the task becomes too high to yield benefits. Conversely, if the testing is too easy— for instance, when the information is already mastered—the benefits are limited. This is because the memory traces cannot be further strengthened through the dual processes of retrieval and re-encoding. Therefore, our model provides a mechanistic rationale for the desirable difficulty hypothesis, linking it to the demands placed on working memory during retrieval practice. The optimal level of difficulty, as suggested by our model, is reached when the task demands moderately engage the WMC, allowing for both retrieval success and additional processing.

In conclusion, the subject of how individual differences affect the testing effect has produced mixed and often hard-to-interpret results. However, these inconsistent findings offer valuable opportunities to understand the general mechanisms behind the testing effect. In this paper, we introduce a theoretical model^1^ that seeks to clarify these discrepancies by examining the interplay between WMC and WM demands. We substantiate our model with evidence from fMRI and EEG studies, showing its consistency with the empirical findings discussed in this review. This model has the potential to illuminate educational applications, providing a unique lens through which to view the testing effect as a trade-off in working memory resources. From an educational standpoint, achieving the right balance between an individual’s WMC and task difficulty—akin to the concept of “desirable difficulty” proposed by Bjork^33^ —may be crucial for unlocking the full potential of retrieval practice for diverse students. Future research could take advantage of big data methodologies to explore this potential trade-off in a more systematic and comprehensive manner.
